# Autoantibodies in myasthenia gravis: cluster analysis and clinical correlations

**DOI:** 10.3389/fneur.2025.1537783

**Published:** 2025-02-04

**Authors:** Xupeng Sun, Meijie Qu, Xi Rong, Mingxing Lv, Yunbin Zhao, Yunjun Yan, Lin Liu, Na Sun, Hua Yue, Min Liu

**Affiliations:** ^1^Department of Neurology, Affiliated Hospital of Qingdao University, Qingdao, China; ^2^Jinan Dian Medical Laboratory Co., Ltd., Jinan, China; ^3^Key Laboratory of Digital Technology in Medical Diagnostics of Zhejiang Province, Dian Diagnostics Group Co., Ltd., Hangzhou, China

**Keywords:** myasthenia gravis, antibodies, clinical manifestations, cluster analysis, clinical research

## Abstract

**Objective:**

This study aimed to explore autoantibody clusters and their correlations with clinical features in 644 myasthenia gravis (MG) patients.

**Methods:**

Medical records of 664 MG patients were reviewed. Five autoantibodies (AChR, MuSK, titin, RyR, and LRP4) were selected for cluster analysis. The various clinical manifestations were compared between clusters. Separate association analyses between individual autoantibodies and clinical manifestations as well as among different MGFA subtypes were also performed without prior clustering.

**Results:**

Two separate autoantibody clusters were identified, with significantly different clinical manifestations. Cluster 1 (485 patients) was characterized by higher proportions of RyR-, titin-, and AChR-, while cluster 2 (179 patients) had higher proportions of RyR+, titin+, and AChR+. Cluster 2 patients were older and had elevated QMG scores and odds of complications, particularly hypertension, diabetes, cardiovascular and cerebrovascular diseases, and eye conditions. Individual antibody analysis revealed that male cases were more likely to be AChR+ and titin+, and older age was associated with AChR+, RyR+, and titin+. Among MGFA subtypes, significant differences were detected in AChR, MuSK, titin, complications, thymoma, and hypertension. As MG severity increased from types I to V, AChR+, RyR+, and titin+ proportions peaked at stage IIa. MuSK+ patients were relatively rare and mostly present in the subtype b group. Type b patients had higher MuSK+ prevalence and increased cardiovascular and cerebrovascular disease incidence rates than type a cases.

**Conclusion:**

Overall, cluster 2 features were less favorable to patients. This study provides valuable insights into the clinical and autoantibody profiles of Chinese MG patients.

## Introduction

Myasthenia gravis (MG) is a complex autoimmune disorder arising from antibody-mediated disruption at the neuromuscular junction, causing muscle weakness and fatigability. The pathogenesis of MG is intricately tied to specific autoantibodies that target critical components of the above junction, including acetylcholine receptor (AChR), muscle-specific kinase (MuSK), and lipoprotein receptor-related protein 4 (LRP4) antibodies (1). Acetylcholine receptor antibody positivity was detected in approximately 80% of patients with the generalized form and 50–60% with the oculomotor form, and 30 to 60% of AChR antibody-negative patients had positive MuSK antibodies. Only approximately 19% of patients with double-negative MG were positive for LRP4 (2, 3). However, LRP4 antibodies were less specific than the first two and can be found in 8% of AChR antibody-positive MG (AChR MG), 15% of MuSK antibody-positive MG (MuSK MG), 4% of other neurologic immunizations, and may also be found in the amyotrophic lateral sclerosis (4). Although some antibodies were less specific and not used for routine diagnosis, as demonstrated in a study on SLE by Melissa R. Arbuckle, various pathogenic autoantibodies can be detected in a single patient. The pathogenicity and co-morbidities are slightly altered compared to those of a single antibody. A similar phenomenon was observed in people with myasthenia gravis, although the underlying mechanisms remained unclear at the time. These proteins are colocalized in the postsynaptic membrane, playing a crucial role in neuromuscular transmission and representing a pivotal driver of the disease’s clinical manifestations. In addition, some MG cases have autoantibodies against striated muscle antigens, titin, and ryanodine receptor (RyR), mainly detected in serum samples from patients with thymoma and late-onset MG. As we know, although RyR and titin antibodies are rare in myasthenia gravis patients, they can help in recognizing affected individuals but are not sufficient as separate diagnosis indicators. According to the generalized criteria for myasthenia gravis, the presence of RyR or titin antibodies alone is typically not enough to confirm a diagnosis of myasthenia gravis. Their sensitivities and specificities as markers of thymoma have been discussed, as well as whether such antibodies correlate with MG severity (5–9). This intricate interplay of autoantibodies adds yet another layer to the heterogeneous spectrum of MG, further intensifying the related enigma.

The distinct clinical profiles of these patients, coupled with the variability in antibodies, raise important questions about the underlying mechanisms of MG and the potential for these autoantibodies to serve as disease markers or even therapeutic targets. AChR antibodies, for instance, the commonest cause of MG, are markers for diagnosis and disease classification in MG patients but not for disease severity (1, 10). Their presence is often associated with clinical features such as fluctuating muscle weakness and fatigue, particularly for the ocular and generalized forms of the disease. MuSK antibodies are detected in approximately 6% of all MG cases, and up to 40% in AChR antibody-negative patients, usually showing severe muscle weakness, facial and bulbar involvement, and resistance to routine treatments (10). MuSK antibody titer appears to correlate with disease severity, in both individual patients and the whole patient population (11, 12). LRP4 plays a central role in synaptic development and maintenance (10). The prevalence of LRP4 antibodies varies greatly among countries (2). Studies indicated LRP4 antibodies are less frequent in the Chinese population compared to Westerners, as they were only found in 1–2.9% of SNMG and 0.8–1.7% of all MG cases, and mostly associated with ocular myasthenia gravis (OMG) (13, 14). Although striated antibodies, including those targeting titin, ryanodine receptor (RyR), actin, myosin, tropomyosin, filamin, and others, are important factors in muscle contraction (15–17), their intracellular localization makes it unlikely for these to play direct pathogenic roles in MG. As G O Skeie mentioned, RyR antibody is found mainly in MG patients with a thymoma MG and correlates with severe MG symptoms (18). A study by Romi et al. similarly showed that, in combination with Osserman typing, the rate of titin positivity correlates with the severity of the disease, and that changes in titin levels can be used as one of the indicators of MG efficacy. Positivity for both RyR and titin suggested a high likelihood of thymoma with a poor prognosis (19). Additionally, Nils Erik Gilhus mentioned that RyR antibody was detected in 70% of MG with thymoma, and its presence was a marker for thymoma and suggested severe MG (20). Nonetheless, titin and RyR antibodies are invaluable prognostic biomarkers (10, 18).This indicates the complex roles of antibodies in MG, and the pressing need to unveil the underlying connections between these autoantibodies and the intricate array of clinical features.

Cluster analysis is a statistical method that groups cases into clusters based on similarities between variables (different autoantibodies produced in this study). However, cluster analysis does not explain the existence of these clusters, and no techniques for determining the reliability and validity of clustering are available. Therefore, the associations of individual autoantibodies with clinical manifestations deserve further attention. East-Asian MG patients differ from Caucasian patients in terms of epidemiological, clinical, immunological, and genetic features (5, 6, 9). In this study, basic information, clinical data, and laboratory findings for five autoantibodies (AChR, MuSK, titin, RyR, and LRP4 antibodies) were collected retrospectively by reviewing the medical records of a representative adult Chinese MG cohort. Through a comprehensive analysis of autoantibody profiles and their associations with diverse clinical features, we aimed to assess the nuanced relationships that shape the clinical challenge of MG. This study enhances our understanding of the disease mechanisms and guides the further development of personalized therapeutic strategies.

## Materials and methods

### Patients

The records of 644 MG patients, diagnosed according to an internationally accepted definition in China, admitted to our clinic between September 2020 and February 2023 were analyzed. Each patient was diagnosed by a clinician with extensive clinical experience, following strict adherence to the typical clinical features, as well as pharmacology, electrophysiology, and serum antibodies. The diagnosis of MG was made based on the following criteria: (a) fluctuating and fatigable muscle weakness and (b) at least two positive results of the following tests: serum antibody assay, neostigmine test, and repetitive nerve stimulation (RNS) test. As for serum antibody titer, AChR-Ab≥0.45 nmol/L or LRP4-Ab≥0.21 nmol/L was defined as positive, considering only AChR+, Musk+, and LRP4+ antibodies. This study followed the Declaration of Helsinki and was approved by the ethics committee of the Affiliated Hospital of Qingdao University (Approval no. QYFYEC2023-66). The data are anonymous, and the information consents were waived. Inclusion criteria were: (1) age above 10 years, with no gender restriction; (2) diagnosis of myasthenia gravis; and (3) detection of five anti-AChR, anti-MuSK, anti-titin, anti-RyR, and anti-LRP4 antibodies. Exclusion criteria were: (1) pregnancy in women, (2) severe cardio-pulmonary insufficiency, (3) serious disease sequelae that affect the ability of daily living, and (4) incomplete clinical data.

### Data collection

This was a cross-sectional retrospective study, in which clinical data were collected by review of medical records input into a clinical database. The recorded data included gender, age at disease onset, MGFA class, disease duration, QMG score, clinical manifestations, and presence of autoantibodies.

### Statistical analysis

SPSS version 26 and R version 4.2.3 were used for data analysis. Using the five antibody profiles, cluster analysis was carried out with the K-modes algorithm for patients with similar autoantibody profiles, which was suitable for categorized data. The similarity between a patient and a cluster center was measured based on the dissimilarity of categorical variables (e.g., Hamming distance). Each patient was then assigned to the cluster with the closest cluster center. Finally, two clusters were selected, and further follow-up analysis was performed according to the frequencies of autoantibodies and clinical features. The chi-square and Fisher’s exact tests were used to compare the frequencies of clinical data and autoantibody characteristics between the two clusters. Associations were assessed using binary logistic regression analysis with the chi-square test and Yates correction, determining odds ratios and 95% confidence intervals (CIs). A *p*-value of <0.05 was considered statistically significant.

## Results

### General features

In total, 664 Chinese MG patients were analyzed. Of these, 352 (53.01%) were females and 312 (46.99%) were males. Onset age ranged from 10 to 92 years, and the mean disease course from disease onset to inclusion was 21 (0.13–480) months. The median QMG score at diagnosis was 8 (0–32). Based on MGFA classification, the highest prevalence was detected in the type IIa group, with 265 (39.91%) cases, followed by the type I group, with 140 (21.08%) cases. The antibody test revealed that 498 (75.00%) cases were positive for AChR, with the highest number. In addition, 343 (51.66%) cases had complications, which were appropriately classified according to disease characteristics. Baseline features, including demographic indices, the prevalence rates of autoantibodies, and clinical manifestations, are shown in Table 1 and Supplementary Table 1.

**Table 1:** Autoantibodies and clinical manifestations of Chinese patients.

Characteristic	Designation	Data	Min-max
Sex	Female	352 (53.01)	
	Male	312 (46.99)	
Age		56 (43, 67)	10–92
QMG score		8 (5, 12)	0–32
Disease duration (month)		21 (6, 52)	0.13–480
MGFA classification	I	140 (21.08)	
	IIIa	75 (11.30)	
	IIIb	66 (9.94)	
	IIa	265 (39.91)	
	IIb	73 (10.99)	
	IVa	10 (1.51)	
	IVb	32 (4.82)	
	V	3 (0.45)	
AChR	N	166 (25.00)	
	P	498 (75.00)	
MuSK	N	653 (98.34)	
	P	11 (1.66)	
RyR	N	594 (89.46)	
	P	70 (10.54)	
Titin	N	485 (72.93)	
	P	179 (27.07)	
LRP4	N	664 (100%)	
	P	0	
Complications present	N	321 (48.34)	
	P	343 (51.66)	
Thymoma	N	562 (84.64)	
	P	102 (15.36)	
Abnormal thymus gland	N	652 (98.2)	
	P	12 (1.8)	
Hypertension	N	513 (77.26)	
	P	151 (22.74)	
Diabetes	N	579 (87.20)	
	P	85 (12.80)	
Malignant tumor	N	636 (95.78)	
	P	28 (4.22)	
Cardiovascular and cerebrovascular diseases	N	597 (89.91)	
	P	67 (10.09)	
Thyroid dysfunction	N	600 (90.36)	
	P	64 (9.64)	
Dermatosis	N	640 (96.39)	
	P	24 (3.61)	
Diseases of the eye	N	643 (96.84)	
	P	21 (3.16)	
Connective tissue disease	N	661 (99.55)	
	P	3 (0.45)	

Categorical variables were presented as n (%). Non-normally distributed continuous variables were presented as Q2 (Q1, Q3). Q1, First quartile; Q2, second quartile; Q3, third quartile; N, negative; and P, positive.

### Autoantibody clusters and their differences in clinical manifestations

**Figure 1:**
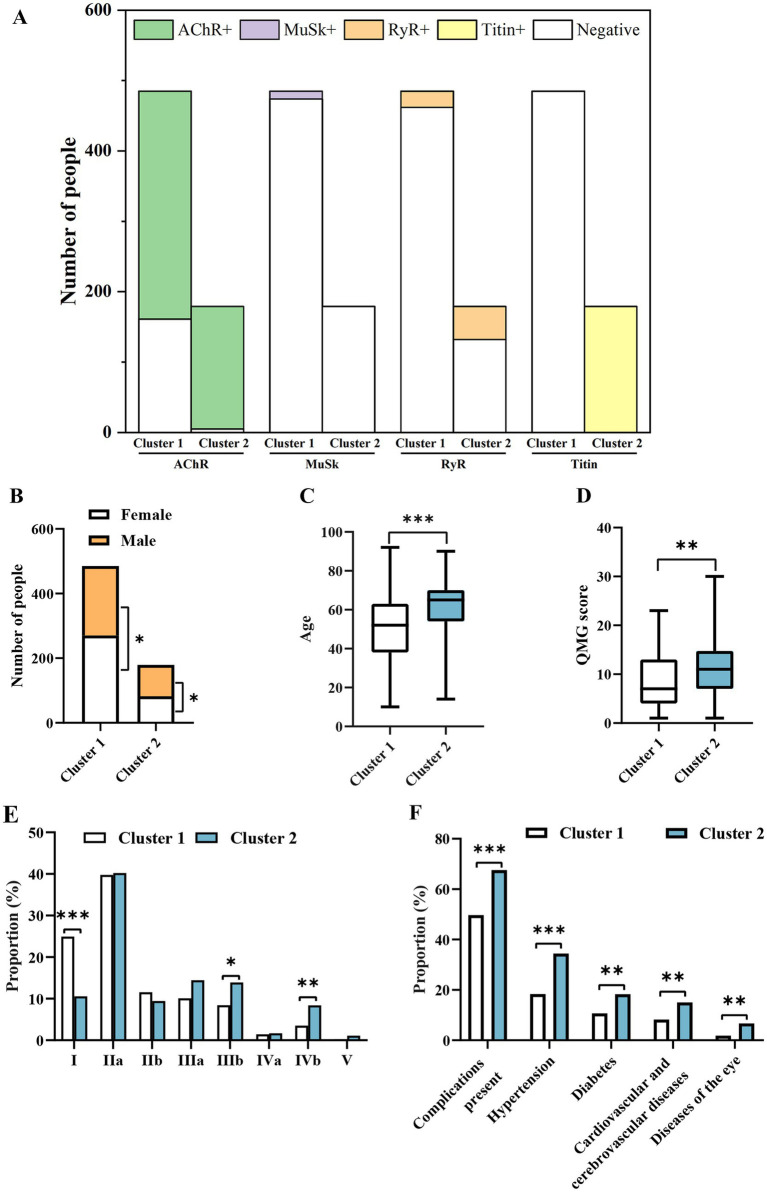
Characteristics of clusters **(A)** and differences in gender distribution **(B)**, age **(C)**, QMG score **(D)**, MGFA subtype **(E)**, and clinical manifestations (F) between the two groups. Only statistically significant differences with a p-value of <0.05 are presented. *p < 0.05, *p* < 0.01, *p < 0.001.

Using cluster analysis, 644 patients were grouped into two distinct clusters based on autoantibodies. The characteristics of the clusters are shown in Figure 1 and Supplementary Tables S1, S2, S4. Subgroup analyses of frequencies of different clinical manifestations in various clusters by age are shown in Supplementary Table S3.

Cluster 1 comprised 485 patients and had higher proportions of RyR- (95.26% vs. 73.74%, *p* < 0.001), titin- (100% vs. 0, *p* < 0.001), and AChR- (33.20% vs. 2.79%, *p* < 0.001) cases compared to cluster 2 (Figure 1A). Cluster 2 included 179 patients and had higher proportions of RyR+ (26.26% vs. 4.74%, *p* < 0.001), titin+ (100% vs. 0, *p* < 0.001), and AChR+ (97.21% vs. 66.80%, *p* < 0.001) cases (Figure 1A). Of note, cluster 2 cases were all positive for titin antibodies, while all cluster 1 cases were negative (Figure 1A). Compared to cluster 2, cluster 1 had a higher frequency of MuSK+ cases (2.27% vs. 0%, *p* = 0.042), but this difference was much smaller compared to the other three antibodies (*p* < 0.001) (Figure 1A). In addition, clustering data after removing MuSK were unchanged, indicating that MuSK had no effects on clustering results.

Cluster 1 exhibited a higher proportion of female patients (55.67% vs. 44.33%, *p* = 0.024), whereas cluster 2 had a higher proportion of males (53.89% vs. 46.11%, *p* = 0.024) (Figure 1B). Compared to cluster 1, cluster 2 displayed significantly higher age at onset (65 vs. 52, *p* < 0.001) (Figure 1C) and QMG score (10 vs. 7, *p* = 0.002) (Figure 1D). In terms of MGFA classification, the proportion of type I cases was higher in cluster 1 than in cluster 2 (24.95% vs. 10.56%, *p* < 0.001), while those of types IIIb (8.45% vs. 13.89%, *p* = 0.035) and IVb (3.51% vs. 8.38%, *p* = 0.009) cases were lower (Figure 1E). The proportion of patients with complications was significantly lower in cluster 1 compared to cluster 2 (49.69% vs. 67.60%, *p* < 0.001) (Figure 1F). Compared to cluster 1, cluster 2 had higher rates of hypertension (18.35% vs. 34.44%, *p* < 0.001), diabetes (10.72% vs. 18.33%, *p* = 0.008), cardiovascular and cerebrovascular diseases (8.25% vs. 15.00%, *p* = 0.009), and eye diseases (1.86% vs. 6.67%, *p* = 0.002) (Figure 1F). These findings suggested that patients positive for AChR, RyR, and titin antibodies were more likely to have these four diseases. Overall, being in cluster 2 was more disadvantageous to patients.

### Associations of individual autoantibodies with clinical manifestations

**Figure 2:**
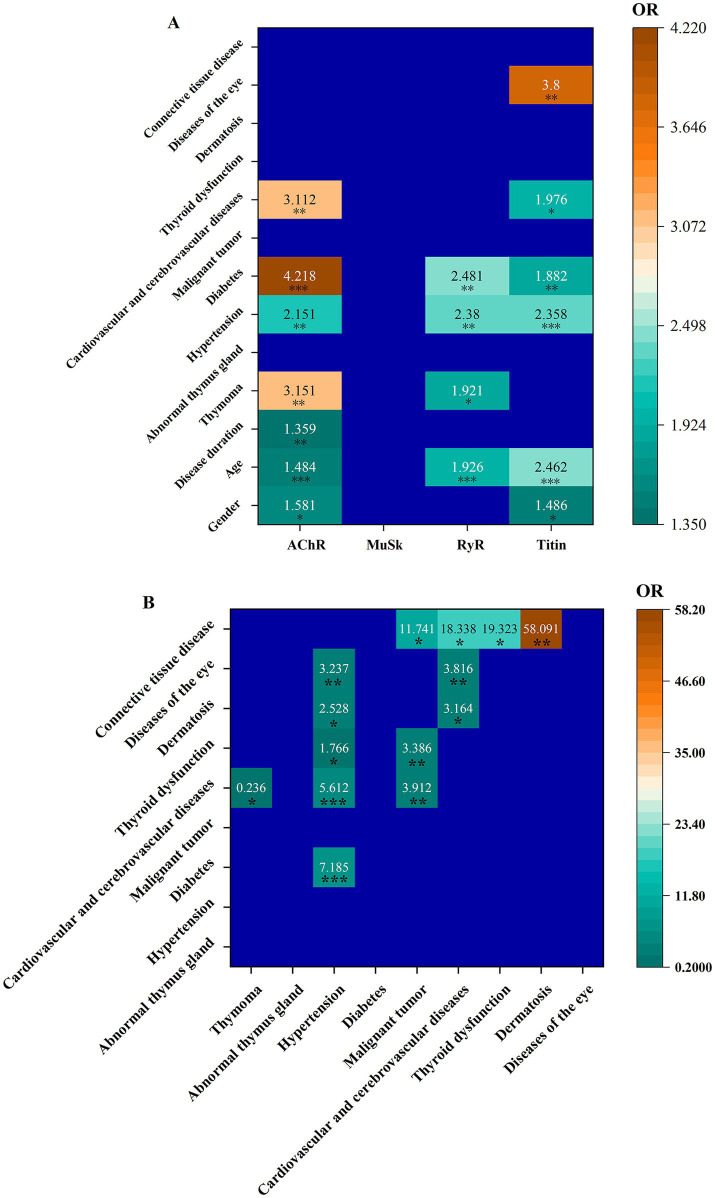
Associations of individual autoantibodies with clinical manifestations **(A)**, as well as among clinical manifestations **(B)**.

The odds ratios (ORs) and 95% CIs for different associations of individual autoantibodies with clinical manifestations are shown in Figure 2A and Supplementary Table S4. In this study, male patients were more likely to be AChR+ [OR = 1.581 (95% CI 1.105, 2.263)], and titin+ [OR = 1.486 (95% CI 1.053, 2.096)]. The older the patient, the higher the risk of being AChR+ [OR = 1.484 (95% CI 1.190, 1.849)], RyR+ [OR = 1.926 (95% CI 1.385, 2.679)], and titin+ [OR = 2.462 (95% CI 1.945, 3.117)]. The longer the disease course, the higher the risk of AChR+ (disease duration was positively correlated with AChR+ [OR = 1.359 (95% CI 1.093, 1.691)]). Similarly, antibody type in the myasthenia gravis was associated with disease complications. AChR+ was positively associated with thymoma [OR = 3.151 (95% CI 1.641, 6.050)], hypertension [OR = 2.151 (95% CI 1.326, 3.490)], diabetes [OR = 4.218 (95% CI 1.906, 9.337)], and cardiovascular and cerebrovascular diseases [OR = 3.112 (95% CI 1.393, 6.950)]. Increased risk of thymoma [OR = 1.921 (95% CI 1.062, 3.474)], hypertension [OR = 2.380 (95% CI 1.414, 4.005)], and diabetes [OR = 2.481 (95% CI 1.359, 4.529)] was found in RyR+ patients. Titin+ patients had increased risk of hypertension [OR = 2.358 (95% CI 1.606, 3.463)], diabetes [OR = 1.882 (95% CI 1.171, 3.026)], cardiovascular and cerebrovascular diseases [OR = 1.976 (95% CI 1.173, 3.330)], and eye diseases [OR = 3.800 (95% CI 1.573, 9.181)]. No associations of anti-MuSK with any clinical manifestations were detected.

The associations among different clinical manifestations were examined (Figure 2B). ORs and 95% CIs for all associations are shown in Supplementary Table S5. Hypertension was positively associated with diabetes [OR = 7.185 (95% CI 4.426, 11.664)], cardiovascular and cerebrovascular diseases [OR = 5.612 (95% CI 3.321, 9.486)], thyroid dysfunction [OR = 1.766 (95% CI 1.012, 3.081)], dermatosis [OR = 2.528 (95% CI 1.099, 5.813)], and eye diseases [OR = 3.237 (95% CI 1.347, 7.776)]. A malignant tumor was positively associated with cardiovascular and cerebrovascular diseases [OR = 3.912 (95% CI 1.651, 9.268)] and thyroid dysfunction [OR = 3.386 (95% CI 1.380, 8.308)]. Cardiovascular and cerebrovascular diseases were positively associated with dermatosis [OR = 3.164 (95% CI 1.210, 8.270)] and eye diseases [OR = 3.816 (95% CI 1.428, 10.197)]. Connective tissue disease was positively associated with malignant tumor [OR = 11.741 (95% CI 1.032, 133.516)], cardiovascular and cerebrovascular diseases [OR = 18.338 (95% CI 1.640, 205.012)], thyroid dysfunction [OR = 19.323 (95% CI 1.727, 216.141)], and dermatosis [OR = 58.091 (95% CI 5.074, 655.007)]. Cardiovascular and cerebrovascular diseases had a negative association with thymoma [OR = 0.236 (95% CI 0.073, 0.76)]. A comparative analysis of titin and RyR in AChR+ patients, both with and without thymomas, is shown in Supplementary Table S6. The results revealed no significant differences in the frequencies of titin and RyR antibodies between these two groups. In other words, the presence or absence of thymomas in AChR+ patients had no effects on titin and RyR antibodies.

### Differences among MGFA subtypes

**Figure 3:**
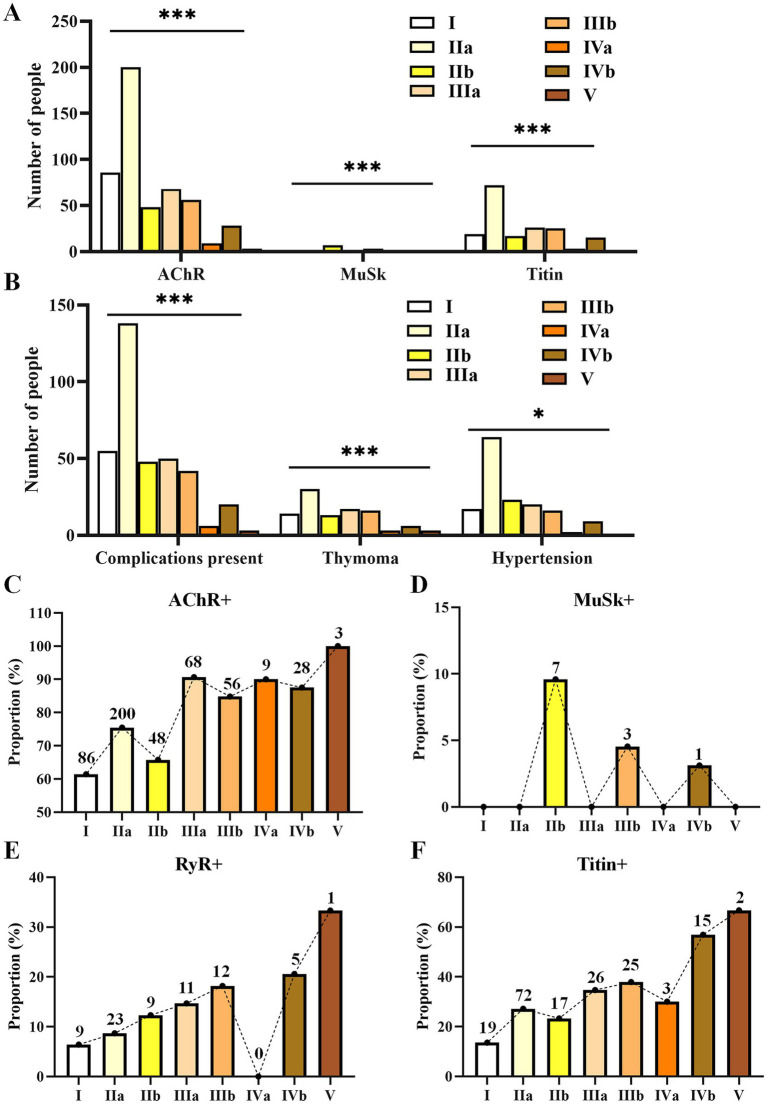
Differences in the numbers of patients with various antibodies **(A)**, complications **(B)**, and alterations in the proportions of antibody positivity **(C–F)** during the progression of MGFA from types I to V. Only statistically significant differences with a *p*-value of <0.05 are presented. *p* < 0.05, *p < 0.01, ***p < 0.001.

Significant differences were found among MGFA subtypes in relation to AChR (*p* < 0.001), MuSK (*p* < 0.001), titin (*p* < 0.001), disease complications (*p* < 0.001), thymoma (*p* < 0.001), and hypertension (*p* = 0.041) (Figures 3A,B; Supplementary Table S7). Alterations in antibodies during disease progression from type I to V are shown in Figures 3C–F. As the disease condition worsened from type I to type V, AChR changes were not significant (Figure 3C). The total number of MuSK+ cases was relatively small (*n* = 11), and all were exclusively distributed in the b-type (IIb = 7, IIIb = 3, IVb = 1); the proportion showed a decreasing trend (Figure 3D). RyR positivity was not found in type IVa, but its proportion showed an upward trend from types I to IIIb and continued to rise in types IVb and V (Figure 3E). The proportion of titin positivity was generally increasing (Figure 3F).

**Figure 4:**
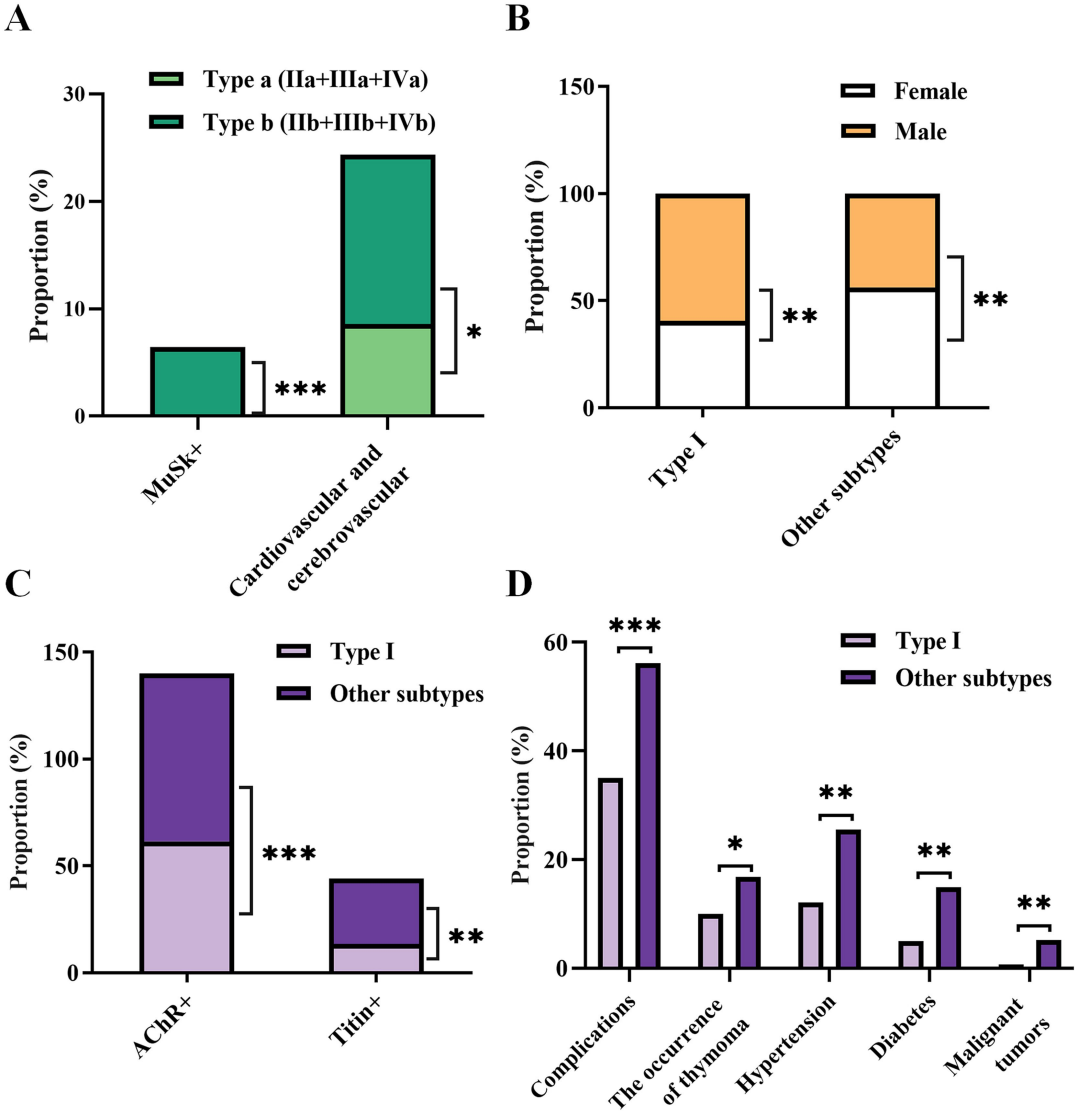
Differences in clinical parameters between the MGFA type a group and type b group **(A)**, as well as between type I and the remaining subtypes **(B–D)**. Only statistically significant differences with a *p*-value of <0.05 are presented. *p < 0.05, **p < 0.01, ***p < 0.001.

Furthermore, an in-depth analysis of differences in multiple clinical parameters between MGFA type a (IIa + IIIa + IVa) and type b (IIb + IIIb + IVb) was performed (Figure 4A; Supplementary Table S8), as well as between type I and the remaining subtypes (Figures 4B–D; Supplementary Table S9). Pronounced differences were found between types a and b with respect to MuSK+ (0 vs. 6.43%, *p* < 0.001) and coexisting cardiovascular and cerebrovascular diseases (8.57% vs. 15.79%, *p* = 0.013) (Figure 4A). In other words, type b cases exhibited higher odds of being MuSK+ and a higher incidence of cardiovascular and cerebrovascular diseases. Compared to the other subtypes, type I had a higher proportion of males and a lower proportion of females (Figure 4B). The proportions of AChR+ (61.43% vs. 78.63%, *p* < 0.001) and titin+ (13.57% vs. 30.53%, *p* < 0.001) cases in other types were higher than in the type I group (Figure 4C). The presence of complications (35.00% vs. 56.11%, *p* < 0.001) and the rates of thymoma (10.00% vs. 16.79%, *p* = 0.048), hypertension (12.14% vs. 25.58%, *p* < 0.01), diabetes (5.00% vs. 14.89%, *p* = 0.002), and malignant tumors (0.71% vs. 5.15%, *p* = 0.02) in other types were also higher than in the type I group (Figure 4D).

## Discussion

To our knowledge, this study first assessed the clinical manifestations of MG patients by cluster analysis to group the patients and to include myasthenia gravis antibody in the analysis into two major categories. In the past, there was only a study to identify disease phenotypes in acetylcholine receptor–antibody myasthenia gravis using proteomics-based consensus clustering. The two subgroups showed huge differences, suggesting that a novel and highly promising classification may exist in our population. Cluster 1 was characterized by higher proportions of RyR-, titin-, and AChR- cases. Cluster 2 had higher RyR+, titin+, and AChR+ cases. Cluster 1 cases were predominantly female, with earlier onset age (52 years), lower QMG scores (7 points), higher prevalence of MGFA Class I, and fewer complications. Cluster 2 cases were predominantly male, with later onset age (65 years), higher QMG scores (10 points), higher prevalence rates of QMG Class IIIb and IVb, and more complications.

Due to exclude MUSK antibodies having no effects on cluster analysis, they were not included in the cluster results.

Although cluster 2 cases had higher QMG scores, corroborating previous studies, AChR IgG concentrations were not directly proportional to disease severity (21). However, a positive correlation was found between AChR IgG concentration and disease course. The proportions of titin+ and RyR+ cases in cluster 2 were higher, especially with titin positivity being 100% in cluster 2. Therefore, this may explain the higher QMG scores detected in cluster 2, indicating severe clinical symptoms (19). In a subsequent analysis of MGFA subtypes, this study also demonstrated that as clinical manifestations become more severe, the positive rates for titin and RyR antibodies increase.

Through separate association analyses of antibodies and complications, titin+, AChR+, and RyR+ cases more likely had thymoma, which is consistent with a previous report (22). In our study, we found that the same systemic damage may be associated with multiple autoantibodies, each of which is different from the others. The patients who had thymoma showed relevance to the AChR and RyR antibodies. Similarly, a retrospective study from the United States, showed that approximately half of the patients with thymoma had more than one type of antibody and the majority of patients had AChR antibodies. All these three antibodies were associated with a higher risk of hypertension and diabetes, possibly related to age, corticosteroid use, or immunosuppression. It had been shown in a number of previous studies that hypertension and diabetes were important risk factors in patients with myasthenia gravis, such as a case–control studies, suggesting that type 2 diabetes mellitus is associated with the onset of late-onset myasthenia gravis. Both past studies and our own research have demonstrated that in MG patients, monitoring of blood pressure and blood glucose should be performed, and early identification and treatment of associated complications should be implemented. Due to the different risk ratios of different age groups, special attention should be given to the elderly, and the correct and effective treatment measures should be identified as early as possible.

In our study, we did not find an association between the antibodies and thyroid dysfunction, abnormal thymus gland, or connective tissue disease. However, several studies have reported inconsistent findings, suggesting that the relationship between myasthenia gravis antibodies and the phenotype of these types of conditions warrants further investigation. Possible reasons for these discrepancies include regional population differences and the relatively weak evidence from observational studies. In the future, more comprehensive studies will emerge, leading to more accurate and reliable conclusions.

Through clustering and correlation analyses of MG patients in the Jiao Dong region of China, this study identified the demographic features and major complications of the local population. This study provided valuable insights into the pathology of myasthenia gravis in this region. We identified two distinct categories, with cluster 2, characterized by RyR+, titin+, and AChR+, representing a more serious disease state. In previous studies, Li Yanfeng found that RyR antibody-positive MG patients had more severe clinical symptoms and their titers correlated with the severity of disease in MG patients with severely diseased thymoma. Romi also mentioned that the rate of titin positivity correlated with the severity of the disease, and changes in titin levels can be used as one of the indicators of MG efficacy (19). We therefore realized that the three antibodies together contribute to disease severity, which was also consistent with the results of our clustering. For a more in-depth mechanism of action, more experiments and larger, multi-location, longitudinal cohorts are expected for validation in the future.

The shortcomings of our study must be acknowledged. The main direction of our exploratory study was to develop a novel grouping of myasthenia gravis. Due to the cross-sectional nature of the clinical data, only representative sub-phenotypes of myasthenia gravis were included. Our study only considered antibodies, future studies should consider an expanded list of clinical variables to provide a better classification (23). Similarly, our study was exploratory, so we could not provide a predictive approach. The study had a relatively small sample size compared to some studies of more common diseases, such as stroke and Alzheimer’s disease. However, given that Myasthenia gravis is a rare disease with a low prevalence, we believe that our study still holds significant value. In fact, the cluster approach provided only a myasthenia gravis snapshot of our cohort at one point in time and with some unavoidable limitations such as bias in information recall, case selection, and a lack of validation in the development and external validation cohorts. The majority of patients in this cohort were yellow, so these may limit applicability to other populations. In the search for accuracy and reliability of the data, we excluded some populations with incomplete information, and the antibody testing was one of the routine and necessary tests in the clinic, so our cohort still showed significant potential for clinical guidance and further exploration of classification.

Overall, in an antibody-confirmed adult myasthenia gravis cohort from China, cluster analysis showed that the diseases of the eye, hypertension, diabetes, cardiovascular, and cerebrovascular diseases occurred with high frequency in cluster2, which was characterized by RyR+, titin+, and AChR+, whereas cluster 1 had a relatively low-frequency occurrence. The results of the subgroup analyses in terms of age showed that eye diseases, hypertension, and diabetes still exhibited significant differences between the two clusters. It was not clear how combinations of these specific antibodies may interact, and further study is needed to determine their contribution to other complications. From a clinical perspective, this finding encourages the consideration of aggressive early treatment of patients with similar autoantibody profiles, regardless of the type of ocular disease. In the future, further studies are needed to validate this finding in larger cohorts, including those with myasthenia gravis who have not yet developed eye diseases and map dynamic predictive models to predict the probability and trajectory of associated diseases.

## Data Availability

The original contributions presented in the study are included in the article/Supplementary material, further inquiries can be directed to the corresponding author.
