# Supplementary material for: Autoantibodies in myasthenia gravis: cluster analysis and clinical correlations
Source: Front Neurol. 2025 Feb 4;16:1537783. doi: 10.3389/fneur.2025.1537783 (PMC11834866; doi:10.3389/fneur.2025.1537783)
Supplement: Supplementary file 1 [file Table_1.docx]

**Supplementary file 1**

**Autoantibodies in** **Myasthenia Gravis: Cluster Analysis, and Clinical Correlations**

**Content**

[**Table S1. Frequencies of different autoantibodies in various clusters 3**](#_Toc187260670)

[**Table S2. Frequencies of different clinical manifestations in various clusters 4**](#_Toc187260671)

[**Table S3. Subgroup analyses of frequencies of different clinical manifestations in various clusters by age. 5**](#_Toc187260672)

[**Table S4. Associations of individual autoantibodies with clinical manifestations 6**](#_Toc187260674)

[**Table S5. Associations among clinical manifestations 7**](#_Toc187260675)

[**Table S6. Differences in titin and RyR in AchR+ patients with and without thymoma 8**](#_Toc187260676)

[**Table S7. Differences among different subtypes of MGFA. 9**](#_Toc187260677)

[**Table S8. Differences between the type a and type b groups 12**](#_Toc187260678)

[**Table S9. Differences between the type I group and all other types 14**](#_Toc187260679)

#

# Table S1. Frequencies of different autoantibodies in various clusters

| **Autoantibodies** |  | **Cluster 1**  **(n = 485)** | **Cluster 2**  **(n = 179)** | **Total** | ***χ^2^*** | ***P*** |
| --- | --- | --- | --- | --- | --- | --- |
| AChR | - | 161 (33.20) | 5 (2.79) | 166 (25.00) | 64.453 | 0.000** |
|  | + | 324 (66.80) | 174 (97.21) | 498 (75.00) |  |  |
| MuSk | - | 474 (97.73) | 179 (100.00) | 653 (98.34) | 4.128 | 0.042* |
|  | + | 11 (2.27) | 0 (0.00) | 11 (1.66) |  |  |
| RyR | - | **462 (95.26)** | 132 (73.74) | 594 (89.46) | 66.173 | 0.000** |
|  | + | 23 (4.74) | **47 (26.26)** | 70 (10.54) |  |  |
| Titin | - | **485 (100.00)** | 0 (0.00) | 485 (73.04) | 664.000 | 0.000** |
|  | + | 0 (0.00) | **179 (100.00)** | 179 (26.96) |  |  |

Categorical variables were presented as n (%). -: Negative, +: Positive. **P* < 0.05, ****P* < 0.001.

# Table S2. Frequencies of different clinical manifestations in various clusters

| **Clinical manifestations** |  | **Cluster 1** | **Cluster 2** | **Total** | ***P*** |
| --- | --- | --- | --- | --- | --- |
| **Gender** | **Female** | **270 (55.67)** | **82 (46.11)** | 352 (53.08) | **0.024*** |
|  | **Male** | **215 (44.33)** | **97 (53.89)** | 312 (46.92) |  |
| **Age** |  | **52.0 (38.0, 63.0)** | **65.0 (54.0,70 .0)**  **77770.0)** | 56.00(43.00,67.00))0)0  00 | **0.000***** |
| Disease duration (month) |  | 24.00(6.00,60.0)0)))  )  )  0660.00) | 18.00(6.00,41.00)) | 21.00(6.00,53.00) | 0.928 |
| **QMG score** |  | **7.00(4.00,12.00)**  **))))**  **(4.00,12.00)** | **10.00(6.00,14.00))0)))** | 8.00(5.00,12.00) | **0.002**** |
| **MGFA classification** | **I** | **121 (24.95)** | **19 (10.56)** | 140 (21.05) | **0.000***** |
|  | IIa | 193 (39.79) | 72 (40.22) | 265 (39.91) | 0.920 |
|  | IIb | 56 (11.55) | 16 (9.44) | 73 (10.98) | 0.454 |
|  | IIIa | 49 (10.10) | 26 (14.44) | 75 (11.28) | 0.110 |
|  | **IIIb** | **41 (8.45)** | **25 (13.89)** | 66 (9.92) | **0.035*** |
|  | IVa | 7 (1.44) | 3 (1.67) | 10 (1.50) | 0.827 |
|  | **IVb** | **17 (3.51)** | **15 (8.38)** | 32 (4.96) | **0.009****  ***** |
|  | V | 1 (0.21) | 2 (1.12) | 3 (0.45) | 0.120 |
| **Complications present** |  | **241 (49.69)** | **121 (67.60)** | 362 (54.67) | **0.000***** |
| Thymoma |  | 69 (14.23) | 33 (18.33) | 102 (15.34) | 0.182 |
| Abnormal thymus gland |  | 10 (2.06) | 2 (1.11) | 12 (1.80) | 0.418 |
| **Hypertension** |  | **89 (18.35)** | **62 (34.44)** | 151 (22.71) | **0.000***** |
| **Diabetes** |  | **52 (10.72)** | **33 (18.33)** | 85 (12.78) | **0.008**** |
| Malignant tumor |  | 22 (4.54) | 6 (3.33) | 28 (4.21) | 0.500 |
| **Cardiovascular andcerebrovascular diseases** |  | **40 (8.25)** | **27 (15.00)** | 67 (10.08) | **0.009**** |
| Thyroid dysfunction |  | 49 (10.10) | 15 (8.33) | 64 (9.62) | 0.504 |
| Dermatosis |  | 16 (3.30) | 8 (4.44) | 24 (3.61) | 0.473 |
| Connective tissue disease |  | 3 (0.62) | 0 (0.00) | 3 (0.45) | 0.292 |
| **Diseases of the eye** |  | **9 (1.86)** | **12 (6.67)** | 21 (3.16) | **0.002**** |

Categorical variables were presented as n (%). Non-normally distributed continuous variables were presented as Q2 (Q1, Q3). Q1: First quartile, Q2: Second quartile, Q3: Third quartile. **P* < 0.05, ***P* < 0.01, ****P* < 0.001.

# Table S3. Subgroup analyses of frequencies of different clinical manifestations in various clusters by age.

| **Clinical manifestations** | **Age group** | **Cluster 1** | **Cluster 2** | **Total** | ***P*** |
| --- | --- | --- | --- | --- | --- |
| **Thymoma** | >=50 | 42(14.74) | 16（10.53） | 58（13.27） | 0.217 |
|  | <50 | 27(13.50) | 17(62.96) | 44（19..38） | **<0.001***** |
| Abnormal thymus gland | >=50 | 2（0.70） | 0（0.00） | 2(0.46) | 0.301 |
|  | <50 | 8(4.00) | 2（7.41） | 10（4.41） | 0.418 |
| **Hypertension** | >=50 | 79（27.72） | 59（38.82） | 138（31.58） | **0.017*** |
|  | <50 | 10(5.00) | 3（11.11） | 13（5.73） | 0.200 |
| **Diabetes** | >=50 | 48(16.84) | 29(19.08) | 77（17.62） | 0.559 |
|  | <50 | 4（2.00） | 4（14.81） | 8(3.52) | **<0.001***** |
| Malignant tumor | >=50 | 17（5.96） | 6(3.95) | 23（5.26） | 0.368 |
|  | <50 | 5（2.50） | 0(0.00) | 5（2.20） | 0.406 |
| Cardiovascular  and cerebrovascular diseases | >=50 | 35（12.28） | 26（17.11） | 61(13.96) | 0.166 |
|  | <50 | 5(2.50) | 1(3.70) | 6(2.64) | 0.714 |
| Thyroid dysfunction | >=50 | 27（9.47） | 15(9.87） | 42（9.61） | 0.894 |
|  | <50 | 22(11.00) | 0(0.00) | 22(9.96) | 0.07 |
| Dermatosis | >=50 | 10(3.51) | 6（3.95） | 16（3.66） | 0.816 |
|  | <50 | 6(3.00) | 2(7.41) | 8（3.52） | 0.244 |
| Connective tissue disease | >=50 | 2（0.70） | 0(0.00) | 2（0.46） | 0.301 |
|  | <50 | 1(0.50) | 0（0.00） | 1（0.44） | 0.713 |
| **Diseases of the eye** | >=50 | 5(1.75) | 11(7.24) | 16（3.66） | **0.004**** |
|  | <50 | 4（2.00） | 1(3.70) | 5(2.20) | 0.571 |

# Categorical variables were presented as n (%). Non-normally distributed continuous variables were presented as Q2 (Q1, Q3). Q1: First quartile, Q2: Second quartile, Q3: Third quartile. **P* < 0.05, ***P* < 0.01, ****P* < 0.001.

# Table S4. Associations of individual autoantibodies with clinical manifestations

| **Clinical manifestation** | **AChR** | **MuSk** | **RyR** | **Titin** |
| --- | --- | --- | --- | --- |
| Gender | **1.581 (1.105, 2.263), 0.012*** | —— | 0.777 (0.470, 1.284), 0.325 | **1.486 (1.053, 2.096), 0.024*** |
| **Age** | **1.484 (1.190, 1.849), 0.000***** | 1.321 (0.628, 2.778), 0.463 | **1.926 (1.385, 2.679), 0.000***** | **2.462 (1.945, 3.117), 0.000***** |
| **Disease duration** | **1.359 (1.093, 1.691), 0.006**** | 1.146 (0.551, 2.386), 0.715 | 0.784 (0.577, 1.065), 0.119 | 0.963 (0.781, 1.118), 0.726 |
| Thymoma | **3.151 (1.641, 6.050), 0.001**** | 1.229 (0.262, 5.772), 0.794 | **1.921 (1.062, 3.474), 0.031** | 1.363 (0.864, 2.149), 0.183 |
| Abnormal thymus gland | 0.661 (0.197, 2.225), 0.504 | —— | 0.768 (0.098, 6.040), 0.802 | 0.537 (0.116, 2.474), 0.425 |
| Hypertension | **2.151 (1.326, 3.490), 0.002**** | 0.335 (0.043, 2.641), 0.299 | **2.380 (1.414, 4.005), 0.001**** | **2.358 (1.606, 3.463), 0.000***** |
| Diabetes | **4.218 (1.906, 9.337), 0.000***** | 0.677 (0.086, 5.359), 0.712 | **2.481 (1.359, 4.529), 0.003**** | **1.882 (1.171, 3.026), 0.009**** |
| Malignant tumor | 1.559 (0.583, 4.169), 0.376 | 2.319 (0.286, 18.772), 0.431 | 1.019 (0.300, 3.466), 0.976 | 0.730 (0.291, 1.830), 0.502 |
| Cardiovascular and cerebrovascular diseases | **3.112 (1.393, 6.950), 0.006**** | —— | 1.791 (0.889, 3.607), 0.103 | **1.976 (1.173, 3.330), 0.011*** |
| Thyroid dysfunction | 1.212 (0.651, 2.254), 0.544 | 2.118 (0.448, 10.024), 0.344 | 1.446 (0.681, 3.070), 0.337 | 0.814 (0.444, 1.491), 0.505 |
| Dermatosis | 2.392 (0.704, 8.124), 0.162 | —— | 2.328 (0.841, 6.443), 0.104 | 1.371 (0.577, 3.262), 0.475 |
| Diseases of the eye | 6.904 (0.919, 51.843), 0.060 | 3.165 (0.386, 25.931), 0.283 | 0.416 (0.055, 3.148), 0.396 | **3.800 (1.573, 9.181), 0.003**** |
| Connective tissue disease | —— | —— | 4.290 (0.384, 47.924), 0.237 | —— |

Results were displayed as OR (95% CI). If OR > 1, the antibody is a risk factor for complications. *p*-value for each association. **P* < 0.05, ***P* < 0.01, ****P* < 0.001.

# Table S5. Associations among clinical manifestations

| **Clinical manifestation** | **Abnormal thymus gland** | **Hypertension** | **Diabetes** | **Malignant tumor** | **Cardiovascular and cerebrovascular diseases** | **Thyroid dysfunction** | **Dermatosis** | **Diseases of the eye** | **Connective tissue disease** |
| --- | --- | --- | --- | --- | --- | --- | --- | --- | --- |
| Thymoma | —— | 0.987 (0.596, 1.635), 0.960 | 0.797 (0.407, 1.561), 0.508 | 1.534 (0.606, 3.882), 0.366 | **0.236 (0.073, 0.766), 0.016*** | 1.308 (0.672, 2.547), 0.430 | 0.491 (0.114, 2.121), 0.341 | 0.916 (0.265, 3.167), 0.890 | —— |
| Abnormal thymus gland |  | 0.304 (0.039, 2.376), 0.256 | 0.615 (0.078, 4.823), 0.643 | —— | —— | —— | 2.486 (0.308, 20.080), 0.393 | —— | —— |
| Hypertension |  |  | **7.185 (4.426, 11.664), 0.000***** | 1.950 (0.880, 4.320), 0.100 | **5.612 (3.321, 9.486), 0.000***** | **1.766 (1.012, 3.081), 0.045*** | **2.528 (1.099, 5.813), 0.029*** | **3.237 (1.347, 7.776), 0.009**** | 6.872 (0.619, 76.318), 0.117 |
| Diabetes |  |  |  | 1.923 (0.756, 4.888), 0.170 | 1.388 (0.696, 2.770), 0.352 | 0.682 (0.285, 1.634), 0.391 | 1.380 (0.460, 4.140), 0.565 | 1.633 (0.536, 4.973), 0.388 | —— |
| Malignant tumor |  |  |  |  | **3.912 (1.651, 9.268), 0.002**** | **3.386 (1.380, 8.308), 0.008**** | 0.987 (0.128, 7.583), 0.990 | 2.498 (0.552, 11.296), 0.234 | **11.741 (1.032, 133.516), 0.047*** |
| Cardiovascular and cerebrovascular diseases |  |  |  |  |  | 2.016 (0.996, 4.081), 0.051 | **3.164 (1.210, 8.270), 0.019*** | **3.816 (1.428, 10.197), 0.008**** | **18.338 (1.640, 205.012), 0.018*** |
| Thyroid dysfunction |  |  |  |  |  |  | 2.591 (0.934, 7.193), 0.068 | 0.986 (0.224, 4.335), 0.986 | **19.323 (1.727, 216.141), 0.016*** |
| Dermatosis |  |  |  |  |  |  |  | 2.971 (0.651, 13.556), 0.160 | **58.091 (5.074, 655.007), 0.001**** |
| Diseases of the eye |  |  |  |  |  |  |  |  | —— |

Results were presented as OR (95% CI). Two clinical manifestations were positively correlated with OR > 1 and negatively correlated with OR < 1. *p*-value for each association. **P* < 0.05 ***P* < 0.01 ****P* < 0.001.

# Table S6. Differences in titin and RyR in AchR+ patients with and without thymoma

| **Subject** | **Designation** |  | | **Total** | **χ^2^** | ***P*** |
| --- | --- | --- | --- | --- | --- | --- |
|  |  | **Without thymoma** | **With thymoma** |  |  |  |
| **RyR** | - | 355 (87.22) | 75 (82.42) | 430 | 1.457 | 0.227 |
|  | + | 52 (12.78) | 16 (17.58) | 68 |  |  |
| Total | | 407 | 91 | 498 |  |  |
| **Titin** | - | 266 (65.36) | 58 (63.74) | 324 | 0.086 | 0.770 |
|  | + | 141 (34.64) | 33 (36.26) | 174 |  |  |
| Total | | 407 | 91 | 498 |  |  |

Cross-analysis of AchR antibody-positivity with and without thymoma (chi square test). Categorical variables were presented by n (%). -: Negative, +: Positive.

# Table S7. Differences among different subtypes of MGFA.

| **Clinical parameters** |  | **MGFA-classification (%)** | | | | | | | | **Total** | ***χ^2^*** | ***p*** |
| --- | --- | --- | --- | --- | --- | --- | --- | --- | --- | --- | --- | --- |
|  |  | **I** | **IIa** | **IIb** | **IIIa** | **IIIb** | **IVa** | **IVb** | **V** |  |  |  |
| Gender | Female | 57 (40.71) | 148 (55.85) | 43 (58.90) | 43 (57.33) | 34 (51.52) | 6 (60.00) | 20 (62.50) | 1 (33.33) | 352 (53.01) | 12.814 | 0.077 |
|  | Male | 83 (59.29) | 117 (44.15) | 30 (41.10) | 32 (42.67) | 32 (48.48) | 4 (40.00) | 12 (37.50) | 2 (66.67) | 312 (46.99) |  |  |
| Total | | 140 | 265 | 73 | 75 | 66 | 10 | 32 | 3 | 664 |  |  |
| **AchR** | - | 54 (38.57) | 65 (24.53) | 25 (34.25) | 7 (9.33) | 10 (15.15) | 1 (10.00) | 4 (12.50) | 0 (0.00) | 166 (25.00) | 35.211 | **0.000***** |
|  | + | 86 (61.43) | 200 (75.47) | 48 (65.75) | 68 (90.67) | 56 (84.85) | 9 (90.00) | 28 (87.50) | 3 (100.00) | 498 (75.00) |  |  |
| Total | | 140 | 265 | 73 | 75 | 66 | 10 | 32 | 3 | 664 |  |  |
| **MuSk** | - | 140 (100.00) | 265 (100.00) | 66 (90.41) | 75 (100.00) | 63 (95.45) | 10 (100.00) | 31 (96.88) | 3 (100.00) | 653 (98.34) | 40.304 | **0.000***** |
|  | + | 0 (0.00) | 0 (0.00) | 7 (9.59) | 0 (0.00) | 3 (4.55) | 0 (0.00) | 1 (3.12) | 0 (0.00) | 11 (1.66) |  |  |
| Total | | 140 | 265 | 73 | 75 | 66 | 10 | 32 | 3 | 664 |  |  |
| RyR | - | 131 (93.57) | 242 (91.32) | 64 (87.67) | 64 (85.33) | 54 (81.82) | 10 (100.00) | 27 (79.41) | 2 (66.67) | 594 (89.46) | 12.879 | 0.075 |
|  | + | 9 (6.43) | 23 (8.68) | 9 (12.33) | 11 (14.67) | 12 (18.18) | 0 (0.00) | 5 (20.59) | 1 (33.33) | 70 (10.54) |  |  |
| Total | | 140 | 265 | 73 | 75 | 66 | 10 | 32 | 3 | 664 |  |  |
| **Titin** | - | 121 (86.43) | 193 (72.83) | 56 (76.71) | 49 (65.33) | 41 (62.12) | 7 (70.00) | 17 (53.13) | 1 (33.33) | 485 (73.04) | 28.404 | **0.000***** |
|  | + | 19 (13.57) | 72 (27.17) | 17 (23.29) | 26 (34.67) | 25 (37.88) | 3 (30.00) | 15 (56.87) | 2 (66.67) | 179 (26.96) |  |  |
| Total | | 140 | 265 | 73 | 75 | 66 | 10 | 32 | 3 | 664 |  |  |
| **Complications present** | - | 85 (65.00) | 127 (47.92) | 25 (36.99) | 25 (41.33) | 24 (37.88) | 4 (50.00) | 12 (37.50) | 0 (0.00) | 302 (48.34) | 27.578 | **0.000***** |
|  | + | 55 (35.00) | 138 (52.08) | 48 (63.01) | 50 (58.67) | 42 (62.12) | 6 (50.00) | 20 (62.50) | 3 (100.00) | 362 (51.66) |  |  |
| Total | | 140 | 265 | 73 | 75 | 66 | 10 | 32 | 3 | 664 |  |  |
| **Thymoma** | - | 126 (90.00) | 235 (88.68) | 60 (82.19) | 58 (77.33) | 50 (75.76) | 7 (70.00) | 26 (81.25) | 0 (0.00) | 562 (84.64) | 32.301 | **0.000***** |
|  | + | 14 (10.00) | 30 (11.32) | 13 (17.81) | 17 (22.67) | 16 (24.24) | 3 (30.00) | 6 (18.75) | 3 (100.00) | 102 (15.36) |  |  |
| Total | | 140 | 265 | 73 | 75 | 66 | 10 | 32 | 3 | 664 |  |  |
| Abnormal thymus gland | - | 138 (98.57) | 258 (97.36) | 72 (98.63) | 74 (98.67) | 65 (98.48) | 10 (100.00) | 32 (100.00) | 3 (100.00) | 652 (98.19) | 2.186 | 0.949 |
|  | + | 2 (1.43) | 7 (2.64) | 1 (1.37) | 1 (1.33) | 1 (1.52) | 0 (0.00) | 0 (0.00) | 0 (0.00) | 12 (1.81) |  |  |
| Total | | 140 | 265 | 73 | 75 | 66 | 10 | 32 | 3 | 664 |  |  |
| **Hypertension** | - | 123 (87.86) | 201 (75.85) | 50 (68.49) | 55 (73.33) | 50 (75.76) | 8 (80.00) | 23 (73.53) | 3 (100.00) | 513 (77.26) | 14.639 | **0.041*** |
|  | + | 17 (12.14) | 64 (24.15) | 23 (31.51) | 20 (26.67) | 16 (24.24) | 2 (20.00) | 9 (26.47) | 0 (0.00) | 151 (22.74) |  |  |
| Total | | 140 | 265 | 73 | 75 | 66 | 10 | 32 | 3 | 664 |  |  |
| Diabetes | - | 133 (95.00) | 225 (84.91) | 65 (89.04) | 61 (81.33) | 57 (86.36) | 9 (90.00) | 28 (71.88) | 3 (100.00) | 579 (87.20) | 12.981 | 0.073 |
|  | + | 7 (5.00) | 40 (15.09) | 8 (10.96) | 14 (18.67) | 9 (13.64) | 1 (10.00) | 6 (28.12) | 0 (0.00) | 85 (12.80) |  |  |
| Total | | 140 | 265 | 73 | 75 | 66 | 10 | 32 | 3 | 664 |  |  |
| Malignant tumor | - | 139 (99.29) | 255 (96.23) | 69 (94.52) | 70 (93.33) | 61 (92.42) | 10 (100.00) | 29 (90.63) | 3 (100.00) | 636 (95.78) | 9.863 | 0.131 |
|  | + | 1 (0.71) | 10 (3.77) | 4 (5.48) | 5 (6.67) | 5 (7.58) | 0 (0.00) | 3 (9.37) | 0 (0.00) | 28 (4.22) |  |  |
| Total | | 140 | 265 | 73 | 75 | 66 | 10 | 32 | 3 | 664 |  |  |
| Cardiovascular and cerebrovascular diseases | - | 130 (92.86) | 242 (91.32) | 62 (84.93) | 68 (90.67) | 55 (83.33) | 10 (100.00) | 27 (84.38) | 3 (100.00) | 597 (89.91) | 9.649 | 0.209 |
|  | + | 10 (7.14) | 23 (8.68) | 11 (15.07) | 7 (9.33) | 11 (16.67) | 0 (0.00) | 5 (15.62) | 0 (0.00) | 67 (10.09) |  |  |
| Total | | 140 | 265 | 73 | 75 | 66 | 10 | 32 | 3 | 664 |  |  |
| Thyroid dysfunction | - | 125 (89.29) | 240 (90.57) | 65 (89.04) | 72 (96.00) | 57 (86.36) | 10 (100.00) | 28 (87.50) | 3 (100.00) | 600 (90.36) | 5.981 | 0.542 |
|  | + | 15 (10.71) | 25 (9.43) | 8 (10.96) | 3 (4.00) | 9 (13.64) | 0 (0.00) | 4 (12.50) | 0 (0.00) | 64 (9.64) |  |  |
| Total | | 140 | 265 | 73 | 75 | 66 | 10 | 32 | 3 | 664 |  |  |
| Dermatosis | - | 133 (95.00) | 256 (96.62) | 70 (95.89) | 72 (96.00) | 65 (98.48) | 10 (100.00) | 32 (100.00) | 2 (66.67) | 640 (96.39) | 10.907 | 0.143 |
|  | + | 7 (5.00) | 9 (3.38) | 3 (4.11) | 3 (4.00) | 1 (1.52) | 0 (0.00) | 0 (0.00) | 1 (33.33) | 24 (3.61) |  |  |
| Total | | 140 | 265 | 73 | 75 | 66 | 10 | 32 | 3 | 664 |  |  |
| Diseases  of the eye | - | 137 (97.86) | 256 (96.60) | 72 (98.63) | 71 (94.67) | 63 (95.45) | 9 (90.00) | 32 (100.00) | 3 (100.00) | 643 (96.84) | 5.524 | 0.596 |
|  | + | 3 (2.14) | 9 (3.40) | 1 (1.37) | 4 (5.33) | 3 (4.55) | 1 (10.00) | 0 (0.00) | 0 (0.00) | 21 (3.16) |  |  |
| Total | | 140 | 265 | 73 | 75 | 66 | 10 | 32 | 3 | 664 |  |  |
| Connective tissue disease | - | 140 (100.00) | 264 (99.62) | 72 (98.63) | 75 (100.00) | 65 (98.48) | 10 (100.00) | 32 (100.00) | 3 (100.00) | 661 (99.55) | 4.240 | 0.752 |
|  | + | 0 (0.00) | 1 (0.38) | 1 (1.37) | 0 (0.00) | 1 (1.52) | 0 (0.00) | 0 (0.00) | 0 (0.00) | 3 (0.45) |  |  |
| Total | | 140 | 265 | 73 | 75 | 66 | 10 | 32 | 3 | 664 |  |  |

# Table S8. Differences between the type a and type b groups

| **Subject** | **Designation** | **Type** | | **Total** | ***χ^2^*** | ***P*** |
| --- | --- | --- | --- | --- | --- | --- |
|  |  | **a** | **b** |  |  |  |
| Gender | female | 197 (56.29) | 97 (56.73) | 294 (56.43) | 0.009 | 0.924 |
|  | male | 153 (43.71) | 74 (43.27) | 227 (43.57) |  |  |
| Total | | 350 | 171 | 521 |  |  |
| AchR | - | 73 (20.86) | 39 (22.81) | 112 (21.50) | 0.259 | 0.611 |
|  | + | 277 (79.14) | 132 (77.19) | 409 (78.50) |  |  |
| Total | | 350 | 171 | 521 |  |  |
| **MuSk** | - | **350 (100.00)** | **160 (93.57)** | 510 (97.89) | 23.000 | **0.000***** |
|  | + | **0 (0.00)** | **11 (6.43)** | 11 (2.11) |  |  |
| Total | | **350** | **171** | 521 |  |  |
| RyR | - | 316 (90.29) | 145 (84.80) | 461 (88.48) | 3.398 | 0.065 |
|  | + | 34 (9.71) | 26 (15.20) | 60 (11.52) |  |  |
| Total | | 350 | 171 | 521 |  |  |
| Titin | - | 249 (71.14) | 114 (66.67) | 363 (69.67) | 1.089 | 0.297 |
|  | + | 101 (28.86) | 57 (33.33) | 158 (30.33) |  |  |
| Total | | 350 | 171 | 521 |  |  |
| Complications present | - | 156 (44.57) | 61 (35.67) | 217 (41.65) | 3.743 | 0.053 |
|  | + | 194 (55.43) | 110 (64.33) | 304 (58.35) |  |  |
| Total | | 350 | 171 | 521 |  |  |
| Thymoma | - | 300 (85.71) | 136 (79.53) | 436 (83.69) | 3.216 | 0.073 |
|  | + | 50 (14.29) | 35 (20.47) | 85 (16.31) |  |  |
| Total | | 350 | 171 | 521 |  |  |
| Abnormal thymus gland | - | 342 (97.71) | 169 (98.83) | 511 (98.08) | 0.760 | 0.383 |
|  | + | 8 (2.29) | 2 (1.17) | 10 (1.92) |  |  |
| Total | | 350 | 171 | 521 |  |  |
| Hypertension | - | 264 (75.42) | 123 (71.93) | 387 (74.28) | 0.736 | 0.391 |
|  | + | 86 (24.58) | 48 (28.07) | 134 (25.72) |  |  |
| Total | | 350 | 171 | 521 |  |  |
| Diabetes | - | 295 (84.29) | 148 (86.55) | 443 (85.03) | 0.463 | 0.496 |
|  | + | 55 (15.71) | 23 (13.45) | 78 (14.97) |  |  |
| Total | | 350 | 171 | 521 |  |  |
| Malignant tumor | - | 335 (95.71) | 159 (92.98) | 494 (94.82) | 1.745 | 0.187 |
|  | + | 15 (4.29) | 12 (7.02) | 27 (5.18) |  |  |
| Total | | 350 | 171 | 521 |  |  |
| **Cardiovascular and cerebrovascular diseases** | - | 320 (91.43) | 144 (84.21) | 464 (89.06) | 6.143 | **0.013*** |
|  | + | **30 (8.57)** | **27 (15.79)** | 57 (10.94) |  |  |
| Total | | 350 | 171 | 521 |  |  |
| Thyroid dysfunction | - | 322 (92.00) | 150 (87.72) | 472 (90.60) | 2.471 | 0.116 |
|  | + | 28 (8.00) | 21 (12.28) | 49 (9.40) |  |  |
| Total | | 350 | 171 | 521 |  |  |
| Dermatosis | - | 338 (96.57) | 167 (97.66) | 505 (96.93) | 0.458 | 0.499 |
|  | + | 12 (3.43) | 4 (2.34) | 16 (3.07) |  |  |
| Total | | 350 | 171 | 521 |  |  |
| Diseases of the eye | - | 336 (96.00) | 167 (97.66) | 503 (96.55) | 0.950 | 0.330 |
|  | + | 14 (4.00) | 4 (2.34) | 18 (3.45) |  |  |
| Total | | 350 | 171 | 521 |  |  |
| Connective tissue disease | - | 349 (99.72) | 169 (98.83) | 518 (99.42) | 1.568 | 0.211 |
|  | + | 1 (0.28) | 2 (1.17) | 3 (0.58) |  |  |
| Total | | 350 | 171 | 521 |  |  |

Results of the cross-over (chi-square) analysis. Categorical variables were presented as n (%). -: Negative, +: Positive. **P* < 0.05 ****P* < 0.001.

# Table S9. Differences between the type I group and all other types

| **Subject** | **Designation** |  | | **Total** | ***χ^2^*** | ***P*** |
| --- | --- | --- | --- | --- | --- | --- |
|  |  | **Type I** | **Other types** |  |  |  |
| **Gender** | **Female** | **57 (40.71)** | **295 (56.30)** | 352 (53.01) | 10.771 | **0.001***** |
|  | **male** | **83 (59.29)** | **229 (43.70)** | 312 (46.99) |  |  |
| Total | | 140 | 524 | 664 |  |  |
| **AChR** | - | **54 (38.57)** | **112 (21.37)** | 166 (25.00) | 17.427 | **0.000***** |
|  | + | **86 (61.43)** | **412 (78.63)** | 498 (75.00) |  |  |
| Total | | 140 | 524 | 664 |  |  |
| MuSk | - | 140 (100.00) | 513 (97.90) | 653 (98.34) | 2.988 | 0.084 |
|  | + | 0 (0.00) | 11 (2.10) | 11 (1.66) |  |  |
| Total | | 140 | 524 | 664 |  |  |
| RyR | - | 131 (93.57) | 463 (88.36) | 594 (89.46) | 3.183 | 0.074 |
|  | + | 9 (6.43) | 61 (11.64) | 70 (10.54) |  |  |
| Total | | 140 | 524 | 664 |  |  |
| **Titin** | - | **121 (86.43)** | **364 (69.47)** | 485 (73.04) | 16.145 | **0.000***** |
|  | + | **19 (13.57)** | **160 (30.53)** | 179 (26.96) |  |  |
| Total | | 140 | 524 | 664 |  |  |
| **Complications present** | - | **91 (65.00)** | **230 (43.89)** | 321 (48.34) | 19.710 | **0.000***** |
|  | + | **49 (35.00)** | **294 (56.11)** | 343 (51.66) |  |  |
| Total | | 140 | 524 | 664 |  |  |
| **Thymoma** | - | **126 (90.00)** | **436 (83.21)** | 562 (84.64) | 3.922 | **0.048*** |
|  | + | **14 (10.00)** | **88 (16.79)** | 102 (15.36) |  |  |
| Total | | 140 | 524 | 664 |  |  |
| Abnormal thymus gland | - | 138 (98.57) | 514 (98.09) | 652 (98.19) | 0.143 | 0.705 |
|  | + | 2 (1.43) | 10 (1.91) | 12 (1.81) |  |  |
| Total | | 140 | 524 | 664 |  |  |
| **Hypertension** | - | **123 (87.86)** | **390 (74.42)** | 513 (77.26) | 11.341 | **0.001**** |
|  | + | **17 (12.14)** | **134 (25.58)** | 151 (22.74) |  |  |
| Total | | 140 | 524 | 664 |  |  |
| **Diabetes** | - | **133 (95.00)** | **446 (85.11)** | 579 (87.20) | 9.672 | **0.002**** |
|  | + | **7 (5.00)** | **78 (14.89)** | 85 (12.80) |  |  |
| Total | | 140 | 524 | 664 |  |  |
| **Malignant tumor** | - | **139 (99.29)** | **497 (94.85)** | 636 (95.78) | 5.388 | **0.020*** |
|  | + | **1 (0.71)** | **27 (5.15)** | 28 (4.22) |  |  |
| Total | | 140 | 524 | 664 |  |  |
| Cardiovascular and cerebrovascular diseases | - | 130 (92.86) | 467 (89.12) | 597 (89.91) | 1.699 | 0.192 |
|  | + | 10 (7.14) | 57 (10.88) | 67 (10.09) |  |  |
| Total | | 140 | 524 | 664 |  |  |
| Thyroid dysfunction | - | 125 (89.29) | 475 (90.65) | 600 (90.36) | 0.236 | 0.627 |
|  | + | 15 (10.71) | 49 (9.35) | 64 (9.64) |  |  |
| Total | | 140 | 524 | 664 |  |  |
| Dermatosis | - | 133 (95.00) | 507 (96.76) | 640 (96.84) | 0.978 | 0.323 |
|  | + | 7 (5.00) | 17 (3.24) | 24 (3.16) |  |  |
| Total | | 140 | 524 | 664 |  |  |
| Diseases of the eye | - | 137 (97.86) | 506 (96.56) | 643 (96.84) | 0.602 | 0.438 |
|  | + | 3 (2.14) | 18 (3.44) | 21 (3.16) |  |  |
| Total | | 140 | 524 | 664 |  |  |
| Connective tissue disease | - | 140 (100.00) | 521 (99.43) | 661 (99.55) | 0.805 | 0.370 |
|  | + | 0 (0.00) | 3 (0.57) | 3 (0.45) |  |  |
| Total | | 140 | 524 | 664 |  |  |

Results of the cross-over (chi-square) analysis. Categorical variables were presented as n (%). 0: Negative, 1: Positive. **P* < 0.05, ***P* < 0.01, ****P* < 0.001.
